# Explainable Artificial Intelligence for Diagnosis and Staging of Liver Cirrhosis Using Stacked Ensemble and Multi-Task Learning

**DOI:** 10.3390/diagnostics15091177

**Published:** 2025-05-06

**Authors:** Serkan Savaş

**Affiliations:** Department of Computer Engineering, Faculty of Engineering and Natural Sciences, Kırıkkale University, Kırıkkale 71450, Türkiye; serkansavas@kku.edu.tr

**Keywords:** liver cirrhosis, explainable artificial intelligence (XAI), stacked ensemble learning, multi-task learning (MTL), transfer learning, Grad-CAM, cirrhosis staging

## Abstract

**Background/Objectives**: Liver cirrhosis is a critical chronic condition with increasing global mortality and morbidity rates, emphasizing the necessity for early and accurate diagnosis. This study proposes a comprehensive deep-learning framework for the automatic diagnosis and staging of liver cirrhosis using T2-weighted MRI images. **Methods**: The methodology integrates stacked ensemble learning, multi-task learning (MTL), and transfer learning within an explainable artificial intelligence (XAI) context to improve diagnostic accuracy, reliability, and transparency. A hybrid model combining multiple pre-trained convolutional neural networks (VGG16, MobileNet, and DenseNet121) with XGBoost as a meta-classifier demonstrated robust performance in binary classification between healthy and cirrhotic cases. **Results**: The model achieved a mean accuracy of 96.92%, precision of 95.12%, recall of 98.93%, and F1-score of 96.98% across 10-fold cross-validation. For staging (mild, moderate, and severe), the MTL framework reached a main task accuracy of 96.71% and an average AUC of 99.81%, with a powerful performance in identifying severe cases. Grad-CAM visualizations reveal class-specific activation regions, enhancing the transparency and trust in the model’s decision-making. The proposed system was validated using the CirrMRI600+ dataset with a 10-fold cross-validation strategy, achieving high accuracy (AUC: 99.7%) and consistent results across folds. **Conclusions**: This research not only advances State-of-the-Art diagnostic methods but also addresses the black-box nature of deep learning in clinical applications. The framework offers potential as a decision-support system for radiologists, contributing to early detection, effective staging, personalized treatment planning, and better-informed treatment planning for liver cirrhosis.

## 1. Introduction

The fight against diseases that pose a mortal threat to world health has become more possible, especially in recent years, with the adaptation of artificial intelligence (AI) technologies to the health sector. While early diagnosis of all diseases contributes positively to treatment processes, early diagnosis of fatal diseases is one of the most important requirements. Diagnosing such diseases in the late stages means that there is not enough time for treatment and an irreversible path has been taken. Liver diseases also rank high among these fatal diseases.

Liver diseases such as cirrhosis, viral hepatitis, and cancer cause more than two million deaths annually, accounting for 4% of all deaths worldwide. One in twenty-five people dies from liver diseases [1]. Cirrhosis is now the ninth leading cause of death in Europe and Southeast Asia, fifth in the Eastern Mediterranean, and tenth in Africa [2]. Cirrhosis also has a significant impact on global health due to the high number of disability-adjusted life-years (DALYs) it generates, representing the 15th leading cause of DALYs globally [3]. Potential years of life lost may be higher, especially in Europe [4]. Cirrhosis is also an expensive disease, and tens of billions of dollars are spent every year to improve diagnosis and treatment processes. Given all these reasons, there is a need for innovative solutions in early detection systems for liver cirrhosis, one of the deadliest diseases.

Different types of data are used to diagnose liver cirrhosis. Among these, clinical findings require examining the patient’s history, such as alcohol use, hepatitis infections, autoimmune diseases, medication use, physical examination, abdominal swelling, jaundice, examination of spider vein-like structures in the skin, and palpation of liver size. Laboratory tests include blood tests, liver function tests, coagulation tests, platelet count, ammonia level, and hepatitis markers. In a biopsy examination, a sample of liver tissue is taken to make a definitive diagnosis of the degree of fibrosis. Today, with the development of non-invasive methods such as elastography, biopsy is less common. Genetic and metabolic tests and/or non-invasive scores are also sometimes performed. In addition to all these, the other diagnostic techniques that have recently become widespread are imaging methods. Techniques such as ultrasound, elastography, magnetic resonance imaging (MRI), computed tomography (CT) scans, and endoscopy can be used to diagnose and grade the disease [5,6,7,8]. Together, these data can be used to diagnose liver cirrhosis, revealing both the cause of the disease and the level of progression.

Especially in the last quarter century, AI techniques have started to be used in the field of health, as in many different disciplines. One of the most common areas of use is disease diagnosis systems. Machine learning (ML) and deep learning (DL) methods, a sub-field under the umbrella of AI, achieve successful results in many different diseases. In this sense, significant contributions are being made to both minimize the effects of diseases that may result in fatal consequences and reduce the costs of diseases with early diagnosis systems. When the specified data of cirrhosis disease are analyzed, it is seen that these data are applicable to ML and DL techniques. In the literature, studies aiming to diagnose liver cirrhosis by applying different ML and DL techniques have been conducted. These studies are described in the second section. However, not enough results have been obtained for the diagnosis and treatment of liver cirrhosis, which is still among the fatal diseases, and the research continues.

In this research, a multi-purpose study was conducted to both detect and level the liver cirrhosis disease. The study differs from other studies in the literature as it not only detects the disease but also successfully classifies the stage of the disease. In this study, detection and classification were performed using image data through deep neural networks (DNNs). In addition, the reliability and generalizability of this study were ensured by the method followed in the data preprocessing stages and the support of explainable artificial intelligence (XAI). The research questions (RQs) of this study are as follows:

RQ1: Can liver cirrhosis detection be successfully realized through DNNs?

RQ2: Can the stage of the detected disease be determined through DNNs?

RQ3: Can XAI be applied to MRI images to ensure the reliability of the results?

The answers to these research questions are discussed in the discussion section and evaluated in comparison with the literature. The contributions of this study to the field are as follows:

- Hybrid convolutional neural network (CNN) and stacked ensemble architecture provided more consistent classification performance compared to single model approaches;
- The staging system developed with multi-task learning (MTL) (AUC: 99.7%) can be used as a decision support tool for radiologists;
- The integration of XAI provides a model to meet the transparency requirements of medical AI systems.

### Related Work

Given the number of variables that can influence clinical outcomes, predicting a particular outcome is often difficult for a clinician. Standard clinical risk assessment models implicitly assume that each risk factor is linearly related to clinical outcomes [9]. Risk factors based on manual physician diagnosis include various situations where human errors can negatively affect the diagnostic process. Misdiagnosis can result in problems such as confusing symptoms with other diseases or missing symptoms at an early stage of the disease. A doctor’s lack of experience, errors in observation, and subjective assessment of clinical findings can further complicate the diagnostic process. In addition, incomplete sharing of the patient’s medical history and misinterpretation or reporting of laboratory and imaging results also increase the risk of error. Environmental factors such as intense work tempo, stress, and time constraints may also lead to rapid and erroneous decisions. Lack of multidisciplinary collaboration and inadequate use of technology in this process can reduce diagnostic accuracy. Therefore, the use of AI-supported tools, automation systems, and up-to-date diagnostic protocols is of great importance to minimize these risks.

ML has been applied to various types of data in liver disease research, including clinical, demographic, molecular, radiologic, and pathologic data. The authors of [10] predicted that using ML tools to create predictive algorithms will change the face of clinical practice in hepatology and transplantation. In the literature, ML and DL studies have been conducted for liver cirrhosis for various purposes. Some of these studies have used ML techniques for liver disease detection without focusing on a specific liver disease [11] and classification techniques such as Decision Tree (DT), Linear Discriminant (LD), Support Vector Machine (SVM) Fine Gaussian, and Logistic Regression (LR) algorithms for the prognosis of liver disease. Laboratory parameters of patients with modelable features were used as the dataset. In one study, the LD algorithm showed the highest accuracy rate (95.8%). In a study using ML for the early prediction of liver disease [12], an Artificial Neural Network (ANN) algorithm was used on data obtained from the UCI ML repository, and an accuracy rate of 88.4% was obtained. Similarly, in another study that accessed the data obtained from the UCI ML repository on Kaggle and performed liver disease prediction with algorithms such as LR, k-Nearest Neighbor (k-NN), eXtreme Gradient Boosting (XGBoost), SVM, Gaussian Naïve Bayes (NB), Random Forest (RF), Decision Tree (DT), Gradient Boosting (GB), CatBoost, AdaBoost, and LightGBM. The most successful model was RF (88.55%) [13]. Comparing different ML algorithms, the authors of [14] created a dataset based on Hepatitis C disease. Here, blood donor, suspected blood donor, fibrosis, and cirrhosis categories were marked, and various features were selected. Five ML algorithms, including NB, RF, k-NN, DT, and ANN, were compared in the study. The ANN algorithm outperformed the other algorithms, with an accuracy of 95.50%. Another study used ensemble ML (RF) to classify hepatitis and cirrhosis based on hepatitis C. The results of the study confirmed the usefulness of ensemble ML for hepatitis C and cirrhosis diagnosis prediction [15].

There are also studies focusing directly on liver cirrhosis in the literature. The authors of [16] performed a prediction on the liver cirrhosis dataset using SVM, DT, and RF algorithms, and the best result was obtained by the RF algorithm, with an accuracy rate of approximately 97%. For the prediction of ascite grades in patients with liver cirrhosis using laboratory and clinical data, the authors of [17] used k-NN, SVM, and NN algorithms, and the k-NN algorithm achieved the best result with 94%. The authors of [18] evaluated a DL model to predict hepatocellular carcinoma (HCC) in patients with hepatitis C cirrhosis. In the study, Recurrent Neural Network (RNN) models outperformed traditional LR models, indicating that RNN models can be used to identify patients with hepatitis C virus-related cirrhosis at a high risk of developing HCC for risk-based HCC outreach and surveillance strategies. The authors of [19] proposed a computer-aided cirrhosis diagnosis system for cirrhosis diagnosis based on ultrasound images. The CNN and SVM technique was used in the study, and 90% success was achieved. The authors of [20] integrated two DL algorithms and a CNN and Capsule Network (CN) hybrid to classify cirrhosis liver disease from MRI. Various data preprocessing techniques were used before these operations, and different algorithms were tried for parameter optimization, and 99.3% accuracy was achieved. In a different study using the CNN algorithm with ResNet50 and DenseNet121 architectures, researchers aimed to perform the classification of alcohol-related versus non-alcohol-related cirrhosis on T2-weighted single-slice images [21]. In the study, the ResNet50 model achieved 75% success. Both models were tried in the ensemble, but they could not improve the performance. The authors of [22] compared DL and ML techniques. In the study, an MLP-based DL model was developed for cirrhosis prediction and compared with DT, k-NN, LR, NB, RF, and SVM. The experimental results show that the model has an accuracy of 80.48%. The authors of [23] proposed a DL approach for the classification of cirrhosis diseases using MRI. This study was implemented in two ways, with 26 features and 56 features. The results were 96.6% accuracy with 26 features and 97.6% accuracy with 56 features.

As can be understood from these studies, liver cirrhosis studies are not yet at a sufficient level, and most research lacks innovative AI techniques. These studies in the literature have the following limitations that can be broadly categorized as follows:

- The accuracy results of some studies are low and not generalizable;
- Most studies are limited to specific ML algorithms. Moreover, in different studies using the same dataset, although the same algorithms are used, the performance of different algorithms is high, which limits the reliability and generalizability of the results;
- DL studies have used intensive data preprocessing and parameter optimization;
- The use of transfer learning techniques is extremely limited. This means models are defined for specific tasks. However, one of the goals of DL architectures is to build models that are generalizable and adaptable to multiple problems;
- The Gradient-Weighted Class Activation Mapping (Grad-CAM) XAI application has not yet been realized in this field.

This study overcomes these limitations identified in the literature and provides more robust and generalizable results. Moreover, the obtained results have generalizable properties thanks to cross-validation techniques and optimization methods.

The rest of the paper is structured as follows: The second section describes the methodology proposed and the material used in this research. In the third section, the findings are presented. Section Four discusses the findings of this study in comparison with the existing literature. In the fifth section, the study is concluded.

## 2. Materials and Methods

This section describes the dataset used in the study and provides information about the methodology applied. To ensure the reliability and generalizability of the results of the study, a strategic approach was followed.

### 2.1. Data and Data Preprocessing

The CirrMRI600+ dataset was used in the study, the first dataset specifically designed for liver cirrhosis research [24]. This dataset contains both T1w and T2w MR images. The dataset contains 310 T1 and 318 T2 abdominal MR images from different stages of cirrhosis. These images were manually segmented. This dataset includes scans exhibiting a variety of morphological changes, reflecting the various complexities associated with real-world complications of cirrhosis, such as contour nodularity, hepatic segment atrophy or hypertrophy, ascites, varices, and splenomegaly. This complexity is crucial for training robust and generalizable DL models that perform effectively on unseen data. The dataset also includes a wide range of disease presentations. Thus, it is also compatible with real clinical scenarios. When creating the dataset, the researchers adopted the following strategy [24]:

- Three different scanners (Achieva, Philips (1.5T and 3T) (Amsterdam, Netherlands), and Symphony, Siemens 1.5 T (Erlangen, Germany) scanners with full anonymization protocols) were obtained to maintain heterogeneity in the MRI scans. To address the variability stemming from the use of different MRI scanners, all images were preprocessed using a standard pipeline: the intensity values were min–max normalized to a [0–1] range, image dimensions were resized to 224 × 224 pixels, and formats were unified using Nibabel. While scanner harmonization techniques were not explicitly applied, the robust cross-validation performance suggests that the ensemble architecture and transfer learning strategies provided sufficient generalization. Future studies could explore domain adaptation methods to further mitigate device-dependent biases;
- To ensure image quality, participating radiologists selected and annotated “good enough” images. The rest were excluded.

This study included patients with liver cirrhosis and a non-cirrhotic control group (the healthy folder). It aimed to capture comprehensive samples of liver cirrhosis with different etiologies and stages, emphasizing variability and various complications. The CirrMRI600+ dataset includes metadata on patient age, gender, and scanner type. In terms of sex distribution, the healthy group consisted of 34 females and 21 males, while the cirrhosis group included 128 females and 190 males. The mean age of healthy individuals was 62.78 years (±14.93), and for cirrhotic patients, it was 60.10 years (±13.76). This demographic spread contributes to the diversity of the training data. In addition, MRI images were acquired from three different scanner types (Philips 1.5T, Philips 3T, and Siemens 1.5T), enhancing heterogeneity. The details are provided in Table 1. This demographic spread contributes to the generalizability of the proposed model.

In this research, T2-weighted 2D images in the dataset were used. To use these images, a series of data preprocessing steps was performed. First, the following information can be given about the data downloaded from the database. T2-weighted images were analyzed in the study. The T2 folders in the healthy subjects folder in the database were considered. There are 55 images with the .nii extension in this folder. Files with .nii extension are files belonging to the Neuroimaging Informatics Technology Initiative (NIfTI) format used to store medical imaging data, and these files usually contain imaging sequences. These sequences are volumetric data obtained by medical imaging techniques such as MRI, collected with specific protocols. All 2D slices from each 3D volume were included in the training process to preserve spatial variability and reduce overfitting. No slice selection filtering (e.g., central slice prioritization) was applied. Spatial resampling or alignment was not required, as the dataset had undergone prior quality control by radiologists. The sequences of the images obtained from these healthy subjects were first extracted using the Nibabel library in the Python programming language. Since the study aims to perform a fully automatic detection and leveling process, all sequences are included in the training and testing processes. In this way, it was also aimed to prevent the overfitting problem. As a result of extracting the sequences of the .nii file, a total of 2143 images of healthy subjects were obtained.

Within the Cirrhosis T2-2D folder in the database, the images are divided into three different folders: train, test, and valid. Since the 10-fold cross-validation (CV) technique was applied in this study to ensure the reliability and generalizability of the results, these folders were brought together, the subjects were gathered, and a total of 318 patient folders were obtained. The MetaData file of the data in the database contains tags such as Patient ID, Age, Gender, and Radiological Evaluation. Using the Patient ID and Radiological Evaluation tags, the data were grouped into “1 = mild”, “2 = moderate”, and “3 = severe” folders. As a result of this process, subfolders were created for 131 patients in the mild folder, 114 patients in the moderate folder, and 73 patients in the severe folder. These subfolders contain different numbers of images for all patients. Therefore, the images were grouped in patientID_images_imageNo format. This resulted in 2838 images in the mild folder, 2391 images in the moderate folder, and 1473 images in the severe folder. Table 2 presents the information on the data status as a result of these data preprocessing stages.

**Figure 1:**
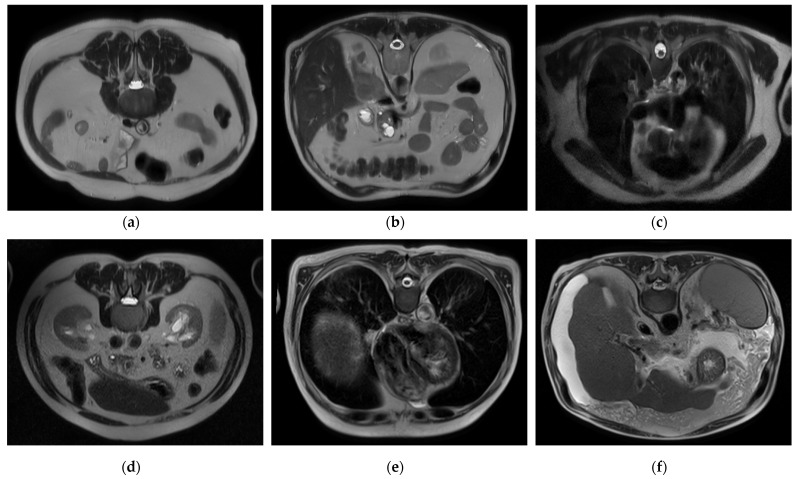
Sample images from dataset: (**a**) healthy (first sequence), (**b**) healthy (mid sequence), (**c**) healthy (last sequence), (**d**) mild, (**e**) moderate, (**f**) severe.

To handle a class imbalance in the binary classification task, a balanced subset was created by randomly selecting 2143 images from the cirrhosis group to match the healthy class. This undersampling strategy ensured balanced class distribution without introducing synthetic data. For the staging task involving mild, moderate, and severe categories, no artificial oversampling or data augmentation was applied. This was a deliberate methodological choice to restrict the model’s learning exclusively to real, clinically validated MRI slices. While this may affect minority class performance, it also avoids introducing synthetic variability that may compromise clinical interpretability. Future work may investigate augmentation techniques as a means of improving severe-class representation. Sample images for each class and level are presented in Figure 1, respectively.

### 2.2. Hybrid CNN, Stacked Ensemble, and XAI-Based Multilayer AI Approach for Automatic Cirrhosis Diagnosis and Stage Classification

In the study, four different tasks were performed, and a fully automated, comprehensive diagnosis and diagnostic process for cirrhosis was created. These tasks are as follows:

- Classification of healthy individuals and individuals with cirrhosis based on image data;
- Automatic detection of the stage (e.g., mild, moderate, or severe) of individuals with cirrhosis;
- Learning the distinctive features of cirrhosis stages with MTL and deep transfer learning (DTL) approaches and improving the classification performance specific to these stages;
- Using XAI techniques to justify the diagnostic decisions made by the model and increase the reliability of the model.

In Task 1, a hybrid approach for classifying the stages of liver cirrhosis in medical images is presented. The method consists of a multilayered pipeline that includes feature extraction with DTL (VGG16, MobileNet, and DenseNet121), combining these features to form a stacked ensemble structure and using XGBoost as a meta-level classifier. Stacked ensemble learning is an ML approach that aims to create a more powerful learning system by combining the insights of multiple models [25]. In this structure, there are usually two layers (a base layer and a meta-layer). The base layer consists of models that operate on different features or representations. In this study, we use DTL models to obtain pre-trained robust representations. The meta-layer is an algorithm that combines the predictions of the base layer models to produce the final predictions. Here, the XGBoost ensemble learning algorithm was chosen as the meta-layer. XGBoost is characterized by its capacity to control overlearning while improving prediction accuracy [26]. This structure combines the powerful representation learning capabilities of DTL and the efficient generalization power of XGBoost to provide high performance on both complex datasets and limited data environments. The stacked ensemble procedure is as follows: “Base models (CNNs) extract high-level features from images, the extracted features are combined to create a meta-feature vector, and the meta-model (XGBoost) performs the final classification on this vector”.

In the stacked ensemble structure, three pre-trained CNN architectures (VGG16, MobileNet, and DenseNet121) were used as feature extractors, each initialized with ImageNet weights (include_top = False). Feature maps were obtained from the last convolutional layers of each model and then flattened into 1D vectors. These flattened feature vectors were concatenated along the feature dimension to form a single composite feature vector for each image. No additional feature normalization or decorrelation techniques (e.g., z-score scaling or PCA) were applied, as the pre-trained models include internal batch normalization, and the chosen XGBoost meta-classifier is robust to scale differences and performs its own feature selection and regularization. The meta-model was trained on these concatenated vectors to perform the final classification. While the relative contribution of each CNN model to the ensemble output was not explicitly analyzed in this study, this is a promising area for future ablation and explainability work.

To assess the generalizability of the model, a 10-fold CV was applied, and the statistical reliability of the results (mean ± standard deviation) was reported. To prevent overtraining and enhance model generalization, several strategies were implemented during the training process. First, an early stopping mechanism was employed based on validation loss monitoring, with a patience parameter of 10 epochs. This ensured that training was halted if the model ceased to improve on the validation set, thereby avoiding unnecessary weight updates and overfitting. Second, a 10-fold CV approach was utilized, whereby the model was trained on 90% of the data and evaluated on the remaining 10% across folds. Although no external held-out test set was used, the validation subset in each fold functioned as an unseen test partition for performance estimation. This methodology enabled robust evaluation of model generalization across diverse data splits and minimized the risk of performance inflation due to overfitting.

**Figure 2:**
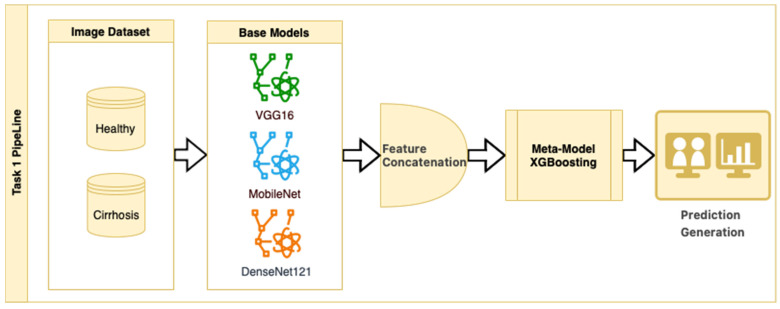
Pipeline of the first task.

This strategy is an example of a heterogeneous stacked ensemble that aims to optimize performance by exploiting the synergy of transfer learning and stacking, especially when working with limited medical data. The pipeline for Task 1 is presented in Figure 2.

Figure 2 shows that pre-trained models in ImageNet, such as VGG16, MobileNet, and DenseNet121, are used only as feature extractors by removing the last layers. These models were used to extract high-level features (edges, textures, shapes, etc.) from images. Within the scope of multi-model integration (ensemble learning), features extracted from three different CNN models are combined to form a single feature vector. Since each model captures different features in this method, the combination provides a stronger representation. A gradient boosting algorithm was trained on the extracted features using the XGBoost Classifier as the meta-model. The XGBoost algorithm is effective in learning non-linear relationships on the features extracted by CNNs and is preferred because it is resistant to overfitting. In addition, the class distribution was preserved while dividing the dataset into 10 parts with a 10-fold CV. Statistical reliability was ensured by calculating the Mean and Standard Deviation over the 10-fold results. Accuracy, precision, recall, and F1-score were calculated for each class, and a confusion matrix and accuracy graph were obtained. To maintain the balance between classes while performing these operations, since the number of images in the healthy class is 2143, 2143 images from the cirrhosis class were also used by the random selection method. It should be noted that random sampling was only applied in the binary classification task to ensure a class balance between healthy and cirrhosis images. For the cirrhosis staging task, no data were discarded: all available images from the mild, moderate, and severe stages were included. Although class imbalance remains a challenge, especially for the severe class, this strategy ensured maximum use of the dataset’s diagnostic variety. Future studies may explore complementary balancing techniques such as class weighting or augmentation.

Within the scope of Task 2, a comprehensive stage detection was performed using the TensorFlow Keras Applications module with a transfer learning technique. In this module, various pre-trained model architectures are available, including ConvNeXt, DenseNet, EfficientNet, EfficientNet, EfficientNet_V2, Inception_ResNet_V2, Inception_V3, MobileNet, MobileNet_V2, MobileNet_V3, NASNet, ResNet, ResNet_V2, VGG16, and Xception. For each module, there are different pre-trained models with different numbers of layers (functions/models). In this study, the base model of each module was transferred and used. Thus, successful models provide a basis for future analysis where more complex models can be explored with additional layers from the same successful module. The models used in the study are listed in Table 3, which provides an overview of the different pre-trained architectures used in the transfer learning process.

As shown in Table 3, 14 CNN architectures were compared using the 10-fold CV technique. The models were initialized with ImageNet weights, and transfer learning was applied by freezing the convolution layers. All models had the same classifier scheme (GlobalPooling → Dropout(0.5) → Dense(3, softmax)). A fixed input resolution (224×224) and batch size (32) were used as standard evaluation protocols. Adam optimization (LR = $1\;\times \;10^{-4}$) and categorical cross-entropy loss were applied. Accuracy and loss values were calculated, and confusion matrices of the models were created. Training curves and metrics were recorded for 100 epochs for each fold.

An MTL process was implemented for Task 3. MTL is an ML approach that allows multiple related tasks to be learned simultaneously. This method aims to increase the generalization capability of the model by sharing common features between different tasks [27]. In contrast to traditional single-task learning approaches, MTL increases generalization ability by encouraging knowledge sharing across related tasks. This approach is particularly advantageous for tasks that require learning common representations. Especially in DL models, MTL produces multiple outputs using the same underlying network structure, thus eliminating the need to train separate models for each task. This approach can improve performance even when source data are limited [28]. One of the most important advantages of MTL is the increase in learning efficiency through knowledge transfer between tasks. For example, by performing object recognition and segmentation tasks simultaneously, an image classification model can achieve better results in both tasks [29]. Another advantage of MTL is that it improves data efficiency. Sharing knowledge from related tasks can improve the performance of the model, especially when labeled data are limited. It helps discover relationships between tasks by learning a common representation and ensures that the transferred knowledge contributes to the learning process of each task. Furthermore, MTL reduces the risk of overfitting, making the model more robust, more balanced, and generalizable. This offers an important advantage, especially when working on small datasets [30]. Due to these properties, MTL is widely used in various fields, such as image processing and biomedical data analysis.

When combined with DTL, MTL becomes even more powerful. While transfer learning transfers the knowledge of a pre-trained model to a new task, MTL provides a more efficient learning process by sharing this knowledge across multiple tasks [31]. In particular, models trained on large-scale datasets (e.g., VGG16, MobileNet, etc.), when combined with MTL, make it possible to achieve high performance with fewer data [32]. This combination saves time and allows the model to produce more consistent results across multiple tasks.

Within the scope of MTL, this study first performed triple classification on the same data and then applied binary classifications for each cirrhosis stage. The VGG16 model was preferred for this task, and the rationale is presented in the Results section. The implementation procedure was as follows:

- Multi-classification: 1_mild ‖ 2_moderate ‖ 3_severe=main output
- Binary classification: 1_mild ‖ others(2_moderate ‖ 3_severe)=mild output
- Binary classification: 2_moderate ‖ others(1_mild ‖ 3_severe)=moderate output
- Binary classification: 3_severe ‖ others(1_mild ‖ 2_moderate)=severe output

The “main output” here is the multiclass classification task, where the model mainly tries to distinguish between three stages (mild, moderate, and severe) simultaneously. This contributes to the identification of the stages of cirrhosis, referred to as “task 2”. The total loss value of this study was calculated using Equation (1):

(1) $$total_{loss}=(1.0\times main_{output_{loss}}+0.5\times mild_{output_{loss}}+0.5\times moderate_{output_{loss}}+0.5\times severe_{output_{loss}})$$

The main task, the classification of cirrhosis stages, is a multiclass classification problem with mutually exclusive classes, where each image belongs to only one class (e.g., mild, moderate, or severe). In such tasks, the model is expected to probabilistically predict the correct class for each instance. In this context, categorical cross-entropy is used as the appropriate cost (loss) function for the main task. This function measures the deviation between the predicted class distribution and the true distribution (usually one-hot coded) and evaluates the distance between probabilities with a logarithmic measure [33]. The categorical cross entropy formula is defined, as in Equation (2):

(2) $$L_{CCE\;}=-\sum_{i=1}^{N}\sum_{c=1}^{C}y_{i,c}\log\left({\hat{y}}_{i,c}\right)$$

Here, N is the number of instances, C is the number of classes, $y_{i,c}$ is the one-hot representation of the true labels, and ${\hat{y}}_{i,c}$ is the probability predicted by the model. The Softmax activation function used in the output layer of the model guarantees that the sum of these probabilities is 1 and reflects the confidence level of each class. This method allows the model not only to predict the correct class but also to optimize the confidence in its prediction. Thus, the model can perform more stable and meaningful classifications in multiclass tasks [34]. The results of the study were trained for 50 epochs using the 10-fold CV method, evaluated with different metrics, and contributed to the correct detection of each stage of cirrhosis.

In Task 4 (the last task), the study applied XAI. The Grad-CAM method, developed to better understand the decision mechanisms of DL models, allows for visualizing which image regions the model focuses on when making a particular prediction [35]. This method is particularly used to explain the decisions of DL-based models, such as CNNs, and understand which features the model considers in the classification process. Grad-CAM uses the activation maps in the final convolution layer of the model to identify the regions that contribute the most to the prediction of the class of interest. First, the derivatives of the model output for a given class are taken to calculate the importance of the feature maps in the final convolution layer. Then, using these importance ratings, the feature maps are weighted, and a class-specific heatmap is obtained. Finally, this heatmap is overlaid on the original image to visualize which regions the model considers when making predictions.

In this study, the Grad-CAM method was used to describe the predictions of the VGG16-based model. After each k-fold CV step of the model’s training process, heat maps obtained by applying Grad-CAM on a sample from the validation set were recorded. Thus, it was analyzed which image regions the model makes decisions by focusing on. The modeling process was based on the VGG16 architecture previously trained on ImageNet, with the last convolutional layers fixed and only the upper layers reconstructed. The final layers of the customized model consist of a dense layer with smoothing, dropout (0.5), and Softmax activation. The model is compiled with a categorical cross-entropy loss function and the Adam optimization algorithm (LR = $1\;\times \;10^{-4}$) for a three-class problem. The dataset consists of labeled liver images containing three stages of cirrhosis. The images were resized to 224×224 pixels to fit the model and normalized using ImageDataGenerator (pixel value scaling: 1/255). To assess the generalizability of the model, a 10-fold CV was applied in this task, too. In each fold, smart data splitting (flow_from_dataframe) was used for training and validation, and the training process was conducted by monitoring accuracy and loss metrics.

The Grad-CAM heatmap ($L_{Grad-CAM}^{c}$) for class c was obtained by weighting each feature map ($A^{k}$) in the relevant layer by the importance coefficients ($\alpha_{k}^{c}$), representing its contribution to the class score and emphasizing positive contributions (Equation (3)) [36]:

(3) $$L_{Grad-CAM}^{c}=ReLU(\sum_{k}\alpha_{k}^{c}\cdot A^{k})$$

where $\alpha_{k}^{c}$ is calculated as the spatial average of the gradients of the score of class c concerning the k-th feature map (Equation (4)) [36]:

(4) $$\alpha_{k}^{c}=\frac{1}{Z}\sum_{i}\sum_{j}\frac{\partial y^{c}}{\partial A_{ij}^{k}}$$

Thanks to this formulation, it is possible to visualize which spatial regions are more effective when the model predicts the class through their positive gradient contributions. The ReLU function ensures that only regions that contribute positively to the class score are considered. In this way, Grad-CAM allows inferences to be made about the model’s internal learning processes and validates the model’s decisions, especially in critical areas such as medical image analysis.

### 2.3. Evaluation Metrics

The correct selection and application of evaluation metrics used in medical research is crucial to objectively assess the performance of models. In this context, not only basic metrics such as accuracy rate but also measures based on loss function, precision, recall/sensitivity, and F1-score were considered. In addition, to comprehensively analyze the classification performance of the model, area under the ROC curve (AUC) values were calculated, and performance comparisons were made within the framework of ensemble learning methods. The evaluation process is based on the true positive (TP), true negative (TN), false positive (FP), and false negative (FN) values for each class.

The accuracy metric used to measure the overall success of the model is calculated as the ratio of correctly predicted instances to the total number of instances, as in Equation (5). A high accuracy rate indicates that the model is generally successful in its predictions for all classes:

(5) $$Accuracy=\frac{TP+TN}{TP+TN+FP+FN}\;$$

Precision refers to the probability that samples predicted as positive are positive and is calculated as shown in Equation (6). It is a critical metric for avoiding false positive predictions and is used to assess the selectivity of the model, especially in imbalanced datasets. A high precision value indicates that the model minimizes false positive errors and is more reliable in its positive classifications:

(6) $$Precision=\frac{TP}{TP+FP}\;$$

Recall (sensitivity) indicates the model’s ability to correctly recognize instances belonging to the positive class. It measures how many true positives in the dataset are correctly predicted. Recall is important in preventing false negatives and is used to determine the effectiveness of the model, especially in critical applications such as medical diagnostics. A high recall value indicates that the model is successful in identifying positive examples. This metric is also called the True Positive Rate (TPR) and is calculated, as in Equation (7):

(7) $$Recall\;(Sensitivity(TPR))=\frac{TP}{TP+FN}\;$$

A metric that balances precision and recall is called the F1-score, which is calculated as the harmonic mean of these two metrics (Equation (8)). It is used to more reliably assess the performance of the model in imbalanced datasets. A high F1-score indicates that the model correctly identifies all instances belonging to the positive class while minimizing false positives. Therefore, it is often a preferred metric to measure the overall classification success of the model:

(8) $$F1\;score=2\times \frac{Precision\;\times \;Recall}{Precision+Recall}\;$$

The AUC—ROC Curve is a method used to evaluate the classification success of the model at different thresholds. The ROC curve shows the relationship between the TPR and False Positive Rate (FPR) of the model, while the AUC value represents the discriminative power of the model. As the AUC value approaches 1, the classification success of the model increases, and as it approaches 0.5, it is understood that the model makes random predictions. AUC is the calculation of the area under the ROC curve (Equations (9) and (10)):

(9) $$FPR=\frac{FP}{FP+TN}$$

(10) $$AUC=\int_{0}^{1}TPR\left(FPR^{-1}\left(x\right)\right)dx$$

Evaluating these metrics together allows the strengths and weaknesses of the model to be identified and provides a more comprehensive analysis of model performance.

### 2.4. Experimental Setup

This study utilized a MacBook M2 Pro with 16 GB RAM, a 16-core GPU, and a 10-core CPU with a 512 SSD for all experimental procedures. The research environment was established using the Anaconda platform and JupyterLab. Python version 3.8.16 served as the primary programming language, complemented by TensorFlow library version 2.16.2, Tensorflow-macOS 2.16.2, and Tensorflow-metal 1.2.0 for DL functionalities. Data visualization and DL tasks were facilitated by Matplotlib (version 3.9.4) and Sklearn (version 1.6.1) libraries, respectively. All experiments were conducted within this computational framework to ensure consistency and the reproducibility of results.

## 3. Results

This section presents the results of the study. The results of the tasks mentioned in the Materials and Methods section are explained.

### 3.1. Binary Classification

First, the task of correctly classifying cirrhosis disease from the images in the dataset was tested. The results of the stacked ensemble method used in this test process are given in Table 4.

The results obtained with the stacked ensemble method for the correct classification of cirrhosis presented in Table 4 comprehensively evaluate how the classification performance varies based on metrics and over different data folds. All performance metrics reported for the binary classification task, including accuracy, precision, recall, and F1-score, were computed separately for each fold in a 10-fold cross-validation framework. The final reported values represent the arithmetic mean across folds. To further enhance statistical interpretability, a 95% confidence interval for the average accuracy was calculated. The mean accuracy was 96.92%, with a standard deviation of ±0.77%, and the 95% confidence interval (CI) was [96.27%, 97.53%]. These statistics confirm the model’s robustness and reliability across independent data partitions. This implies that the model has good generalization ability and performs similarly across different data subsets. In the cross-class performance analysis, the precision value for the healthy class is 95.12%, on average, for the predictions of the healthy class. The low standard deviation (0.0119) indicates a stable performance in this metric. This shows that the model keeps the false positives for the healthy class at a very low level. The average recall rate for the healthy class is 98.93%, which is quite high. This result again shows that the model successfully detects true positives belonging to the healthy class. An F1-score of 96.98% was obtained with balanced high precision and recall values. From these results, it is understood that the stacked ensemble structure model performs well in the healthy class in general. In the cirrhosis class, it was observed that the accuracy level of the model in predicting the cirrhosis class is quite high, with an average precision value of 98.89%. The recall rate for the cirrhosis class is 94.91%, which is slightly lower than the healthy class. This may indicate that some samples belonging to the cirrhosis class were classified as false negatives. A balanced and high performance was achieved in the cirrhosis class, with an average F1-score of 96.85%. There is a balanced performance between the classes, and the weighted averages of the classes weighted by the number of support are the same as the macro average since an equal number of samples were selected. The standard deviation values in all metrics are quite low (0.0072–0.0130). Thus, it can be stated that the stacked ensemble method gives consistent results in different folds, and the model performs reliably.

**Figure 3:**
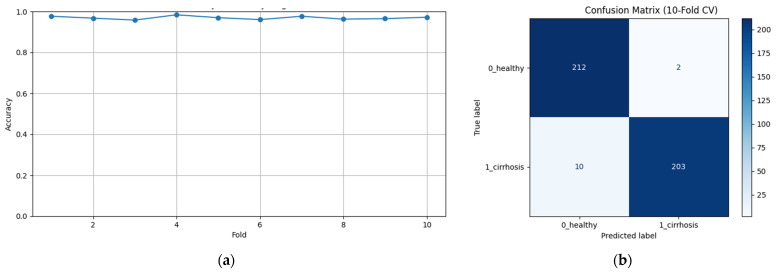
Binary classification: (**a**) 10-fold CV accuracy and (**b**) confusion matrix results.

The stacked ensemble method showed very high and consistent performance in the cirrhosis classification task in both the healthy and cirrhosis classes. The relatively lower recall in the cirrhosis class (94.91%) suggests that the model should pay more attention to false negatives in this class. This can be especially critical in clinical applications. The accuracy graph and confusion matrix obtained in the study are presented in Figure 3, respectively.

As can be seen in Figure 3a, binary classification provides a high level of accuracy for each fold, while the oscillation in the graph is very low, so it can be inferred that consistent results were obtained. In Figure 3b, an average confusion matrix is shown. When the results were analyzed, it was observed that approximately two of the healthy samples were incorrectly predicted, while approximately 10 samples from the cirrhosis class were incorrectly predicted.

### 3.2. Detection of Cirrhosis Stages

Within the scope of the second task in the study, the correct detection of the stages of cirrhosis was performed. In this detection process, images of people with cirrhosis were used, and healthy images were not used. The results of the 14 pre-trained models used for the detection process are presented in Table 5.

Table 5 shows the first, middle, and last values of the 10-fold CV training for each model. To further enhance statistical interpretability and robustness analysis, the standard deviation and 95% CIs were calculated. The average accuracy of the models ranges from 41.84% (EfficientNetB0) to 96.45% (VGG16). The average loss values ranged from 0.1199 (VGG16) to 1.0670 (EfficientNetB0). This reveals that the success of the models in classifying the stages of cirrhosis varies significantly. In particular, evaluating the accuracy and loss values together provides holistic information about the classification accuracy and uncertainty level of the models.

In the overall performance evaluation, four models stand out: VGG16, MobileNet, MobileNetV2, and DenseNet121. VGG16 demonstrated the best performance in terms of average accuracy, achieving 96.45%, with a standard deviation of ±0.71% and a 95% confidence interval (CI) of [95.94%, 96.95%]. Its mean loss value was 0.1199 ± 0.0082, with a 95% CI of [0.1140, 0.1258]. These results indicate that VGG16 is highly effective in classifying cirrhosis stages and minimizing classification errors.

MobileNet achieved a mean accuracy of 95.91% (±1.17%), with a 95% CI of [95.08%, 96.75%], while MobileNetV2 reached 95.69% (±0.90%), with a 95% CI of [95.06%, 96.35%]. The mean loss values were 0.2488 ± 0.0850 for MobileNet (95% CI: [0.1876, 0.3092]) and 0.1962 ± 0.0639 for MobileNetV2 (95% CI: [0.1505, 0.2419]). These findings show that both compact architectures are effective in the classification task.

DenseNet121 achieved a mean accuracy of 94.17%, with a standard deviation of ±0.89% and a 95% CI of [93.53%, 94.81%]. Its mean loss was calculated as 0.1817 ± 0.0423, with a 95% CI of [0.1515, 0.2120]. These results demonstrate that consistent classification performance can also be achieved using deeper architectures.

Overall, these statistics highlight the strong and consistent performance of all four CNN architectures, reinforcing the reliability of the proposed multi-task learning framework for cirrhosis stage detection.

ResNet50V2 and NASNetMobile were found to be moderate performers. ResNet50V2 showed a significant performance, with an accuracy of 93.56% and a loss of 0.3766. However, the relatively high loss value suggests that the classification uncertainties of the model are relatively higher compared to lower-accuracy models. NASNetMobile demonstrated a similar level of success, with 91.84% accuracy and 0.2397 losses. These results suggest that the model offers a reasonable classification performance, but further improvements may be required.

InceptionV3 and InceptionResNetV2 stand out as models with inconsistent performance. InceptionV3 performed acceptably, with an accuracy of 86.17% and a loss of 0.5182, but the loss value indicates that the classification uncertainties of the model are relatively high. InceptionResNetV2 shows a similar trend with 88.31% accuracy and 0.4057 loss. While these models offer reasonable performance in terms of accuracy, they may require optimization to lower the loss values.

**Figure 4:**
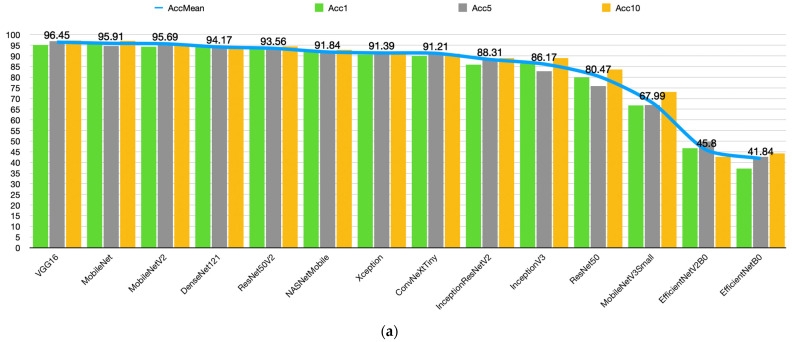

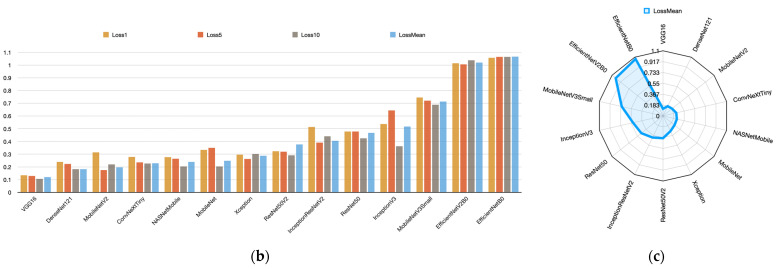
(**a**) Accuracy, (**b**) loss overall, and (**c**) loss average plots of the models.

EfficientNet, EfficientNetV2, and MobileNetV3 were the low-performing models. EfficientNetB0 and EfficientNetV2B0 were far below the others, with low accuracy (41.84% and 45.80%) and high loss (1.0670 and 1.0207). It was concluded that these models were not able to perform appropriate feature learning for this dataset and needed further hyperparameter optimization. MobileNetV3, with an accuracy of 67.99% and a loss of 0.7141%, performed poorly compared to its predecessors, indicating that the model architecture was not well adapted to the classification task on this dataset. Accuracy and loss graphs of the models are presented sequentially in Figure 4.

**Figure 5:**
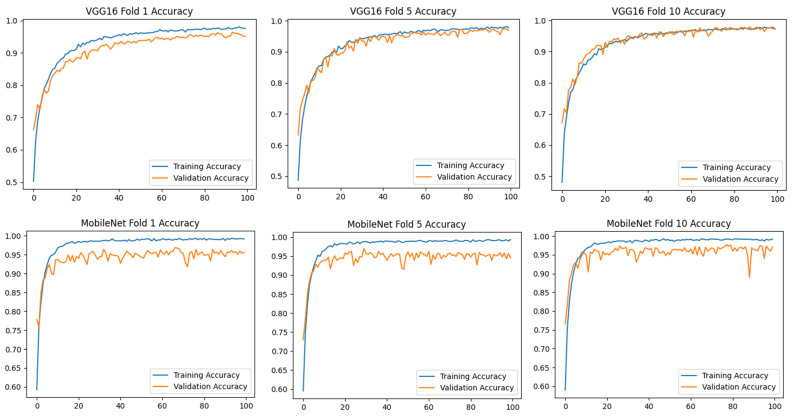

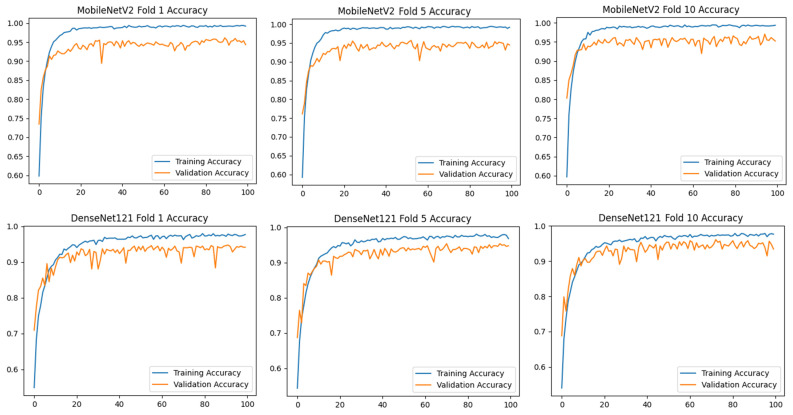
Accuracy graphs of models with high classification success.

Figure 4 shows that models such as VGG16, MobileNet, and DenseNet121 showed superior performance with the highest accuracy and lowest loss values in the classification of cirrhosis stages. In particular, these models provide reliable results by minimizing classification errors. However, for models such as EfficientNet, which perform poorly, model performance can be improved using hyperparameter optimization, data augmentation techniques, or larger datasets. The first-, middle-, and last-fold accuracy graphs of the VGG16, MobileNet, MobileNetV2, and DenseNet121 models, which were found to be the most successful pre-trained models in the study, are presented in Figure 5, respectively.

**Figure 6:**
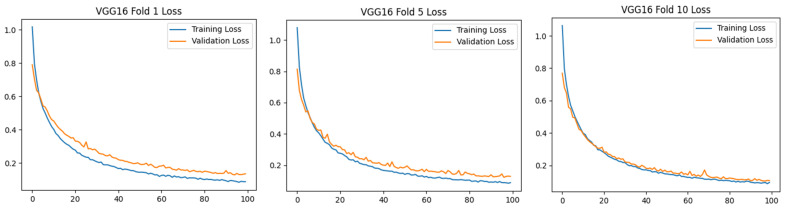

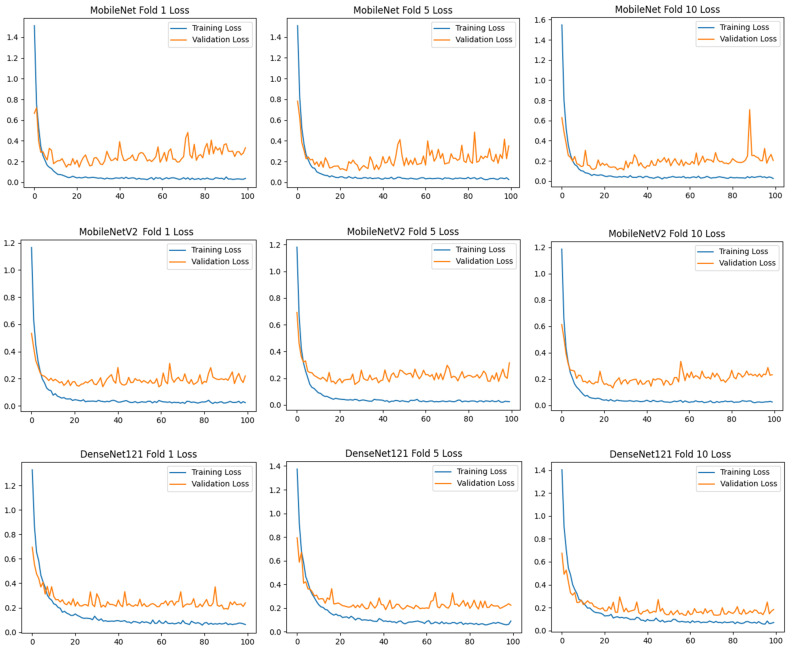
Loss graphs of models with high classification success.

Figure 5 shows that the VGG16 model has a stable training–validation process in all fold trainings. While the MobileNet models achieve high-performance rates, it is seen that the training and validation difference is slightly higher compared to the DenseNet121 model. While the VGG16 and DenseNet121 models increased their training and validation rates over 100 epochs, the MobileNet models did not significantly increase their accuracy rates after about the 30th epoch, but to maintain the standard, training was continued for 100 epochs in these models as well. Loss metric plots for these models were also obtained and are presented in Figure 6.

**Figure 7:**
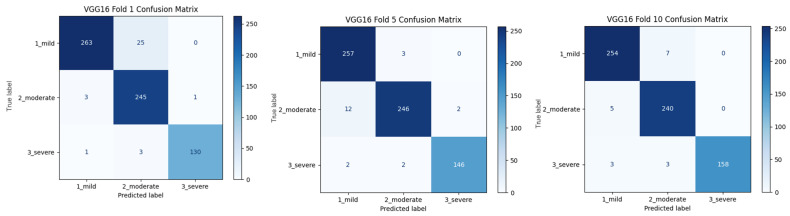

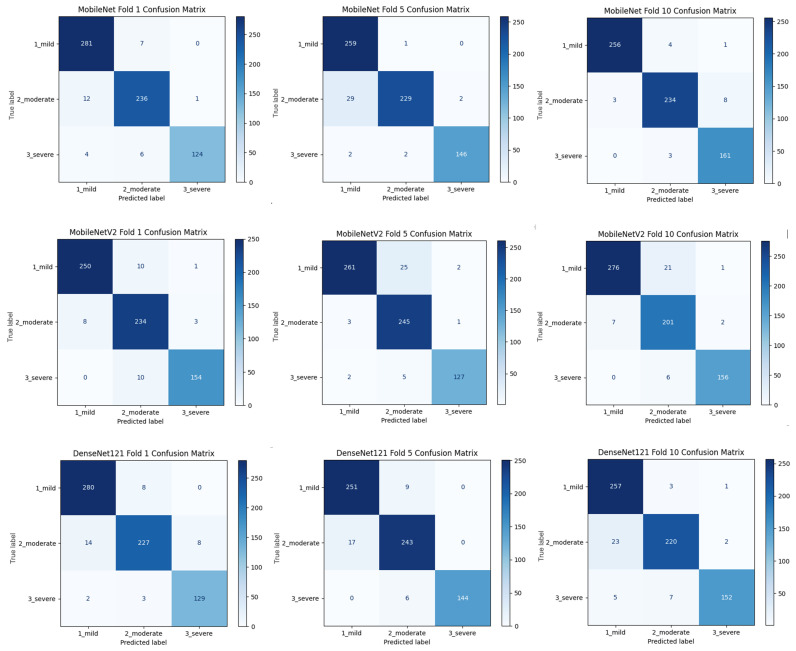
Confusion matrices of models with high classification success.

When Figure 6 is analyzed, the VGG16 model is again the most consistent in terms of loss values. Although the results of the other three models are close to each other, it is observed that the DenseNet121 model generates a more consistent loss graph at Folds 5 and 10. The confusion matrix graphs of these models are presented in Figure 7.

As shown in Figure 7, the confusion matrices of the models with high classification success (VGG16, MobileNet, MobileNetV2, and DenseNet121) for different folding scenarios show the accuracy and error rates of each model by class. In all models, the 1_mild class achieved the highest overall accuracy. However, the predictions in the 2_moderate and especially in the 3_severe class have relatively higher error rates. While the VGG16 model performed quite consistently in the 1_mild class, it made erroneous predictions in some cases in the 3_severe class. Similarly, the MobileNet model seems to be more prone to mispredictions in the 2_moderate class, which is often classified as 1_mild. Among the folds, MobileNet’s performance was observed to be lower than the other folds, especially in Fold 5. MobileNetV2 and DenseNet121 performed better in terms of the balance between classes. While MobileNetV2 generally achieved high accuracy in the 1_mild and 2_moderate classes, it occasionally made incorrect predictions in the 3_severe class. The DenseNet121 model, on the other hand, showed better accuracy in the 2_moderate class compared to the other models and achieved a more balanced classification success across classes. In general, the tendency of models to make errors in the 3_severe class suggests that this class is more difficult to distinguish than the others. This may be due to class imbalance in the dataset or the low discriminative power of the classes.

When Figure 7 is examined, the importance of the 10-fold CV application is once again understood. Different outputs were obtained at different rates in different folds of the study. These results are important for the robustness, consistency, and generalizability of the classification and detection method developed in the research.

### 3.3. Multi-Task Learning

In the MTL task, multidimensional findings were obtained, and the analysis results were evaluated based on the results obtained in four dimensions. The VGG16 model, which was found to be the most successful in the previous layers of the study procedure, was used in the study, and it is possible to test with other models. The accuracy, loss, and AUC rates obtained for MTL classification tasks are given in Table 6.

Table 6 shows that the metrics in the training and validation datasets show generally successful results. For the training dataset, the model demonstrates high consistency, with Main Output Accuracy (MaOAcc) ranging between 95.50% and 96.58%, indicating robust generalization. Moreover, the Main Output Area Under the Curve (MaOAUC) values are almost perfect (99.59–99.75), indicating the distinctive success of the model. The Main Output Loss (MaOLoss) values in the training process are quite low (0.1386–0.1557), indicating that the model has a good generalization capability. Notably, the Severe Output Accuracy (SOAcc) is particularly high (98.32–98.63%), suggesting strong performance in identifying severe cases, while the Moderate Output Accuracy (MoOAcc) shows slightly more variability (91.89–92.95%). Loss values for all categories remain relatively stable, with the highest variability observed in Moderate Output Loss (MoOLoss), ranging from 0.2238 to 0.2363.

**Figure 8:**
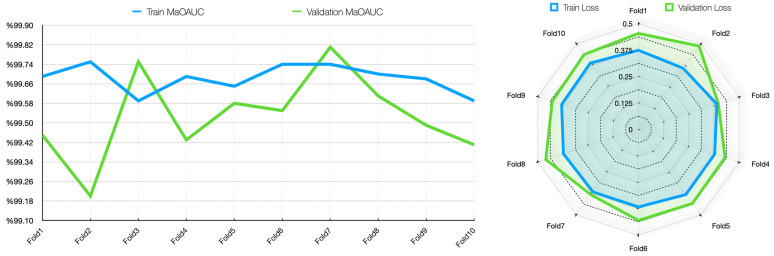
AUC and loss results for MTL.

In the validation dataset, the model maintains strong performance but exhibits greater variability, particularly in Main Output Accuracy (MaOAcc: 93.74–96.71%) and Moderate Output Accuracy (MoOAcc: 89.20–93.58%). The highest MaOAcc (96.71%) and MaOAUC (99.81%) are achieved in Fold 3, suggesting exceptional performance in that fold. However, Fold 2 shows comparatively lower performance in MaOAUC (99.20%) and MoOAcc (89.36%), indicating potential sensitivity to data distribution shifts. The Severe Output Accuracy (SOAcc) remains consistently high (95.46–98.90%), reinforcing the model’s reliability in classifying severe cases. For the validation set, the main output classifier achieved a mean accuracy of 95.07 ± 0.97%, with a 95% CI of [94.37%, 95.76%], indicating a robust and consistent global classification capability. Among the sub-class outputs, the mild stage classifier reached a mean accuracy of 94.11 ± 1.13% (95% CI: [93.30%, 94.92%]), while the moderate stage classifier showed a relatively lower performance with a mean accuracy of 91.15 ± 1.60% (95% CI: [90.01%, 92.29%]), reflecting the difficulty of distinguishing this intermediate stage. Notably, the severe stage classifier achieved the highest accuracy among all subtasks with a mean of 97.56 ± 0.94% (95% CI: [96.88%, 98.23%]), suggesting that advanced cirrhotic patterns are more distinct and easier to identify in MRI images. These results confirm that the proposed multi-task architecture not only maintains high overall accuracy but also provides detailed and clinically meaningful stratification of cirrhosis stages. The losses in the validation set, particularly MoOLoss (peaking at 0.2776 in Fold 2), suggest that moderate cases may present a greater challenge, possibly due to their intermediate nature. However, although the loss values in the validation set (MaOLoss, MiOLoss, and MoOLoss) are slightly higher than the training set, it is understood that the generalization capability of the model is sufficient. The AUC and loss plots obtained in the MTL analysis are presented in Figure 8.

**Figure 9:**
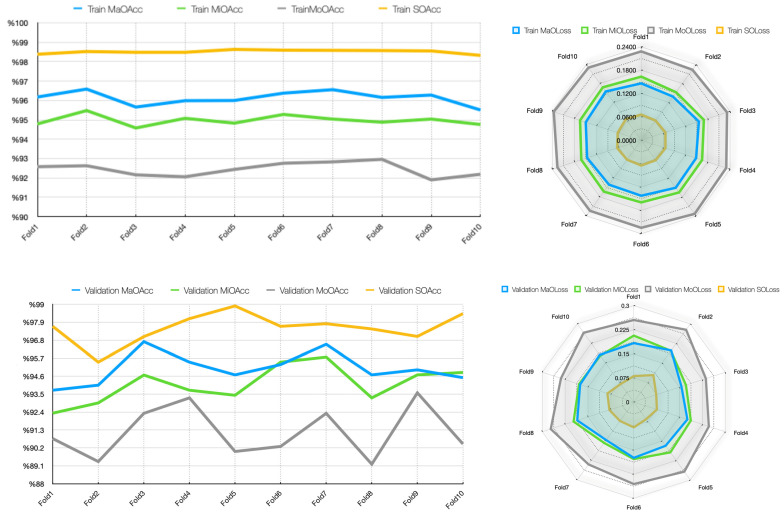
Training and validation accuracy and loss graphs for each task for MTL.

Figure 8 shows that the Main Output AUC (MaOAUC) values are above 99.59% in the training dataset, indicating that the discriminative ability of the model is very high. The validation AUC values also remained generally high, with an excellent performance of 99.81% in Fold 3 and a slight variability, dropping to 99.20% in Fold 2. While the training loss values remained between 0.3581 and 0.3894, indicating a balanced optimization process, the validation losses (0.3819–0.4874) fluctuated slightly more, reaching a peak of 0.4874 in Fold 2. Although this suggests minor generalization problems that may be due to distributional differences in the validation data, the overall low loss and high AUC values indicate that the model is reliable, and there is no obvious overfitting problem. The training and validation accuracy and loss plots for each task for MTL are presented in Figure 9.

**Figure 10:**
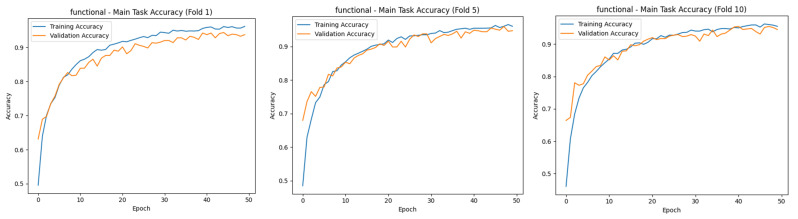

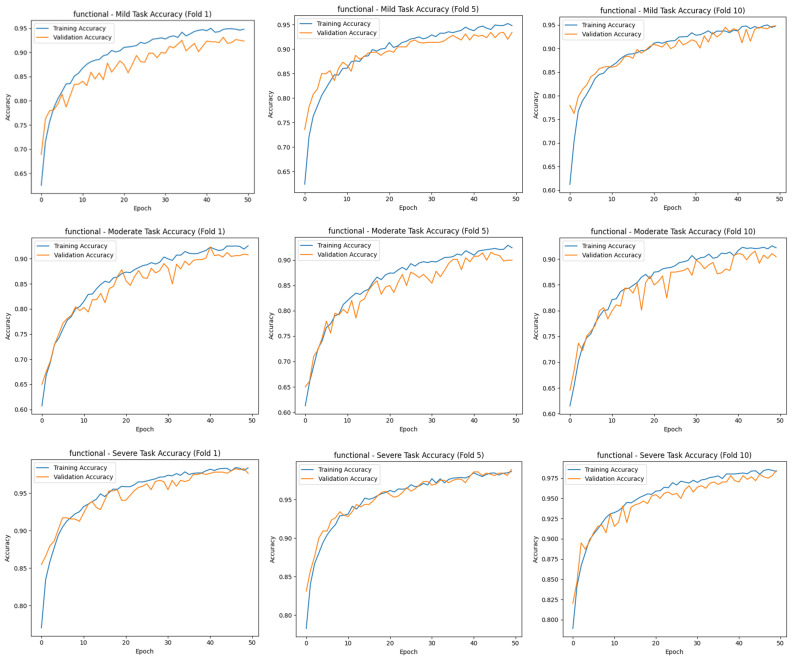
Accuracy graphs for MTL tasks.

When Figure 9 is analyzed, in general, the metrics obtained in both the training and validation phases show that the model performs well at different output levels (mid, moderate, and severe). The low loss rates in training and high accuracy rates in validation show that the model not only adapts to the training data but also successfully demonstrates its generalization capability. These results suggest that the model can be safely used in application environments. Figure 10 shows the first-, middle-, and last-fold training and validation accuracy plots for the MTL task.

Figure 10 visualizes the training and validation accuracies of the MTL model for different tasks (main task, mild, moderate, and severe) at epochs 1, 5, and 10. In all graphs, it can be observed that both training and validation accuracies increase steadily over time and generally reach high accuracy levels by the end of the 50th epoch. Especially for the main task, the accuracy values exceed 90%, and the training/validation curves are very close to each other. This suggests that the model avoids overfitting and shows high generalization success in the overall classification task.

**Figure 11:**
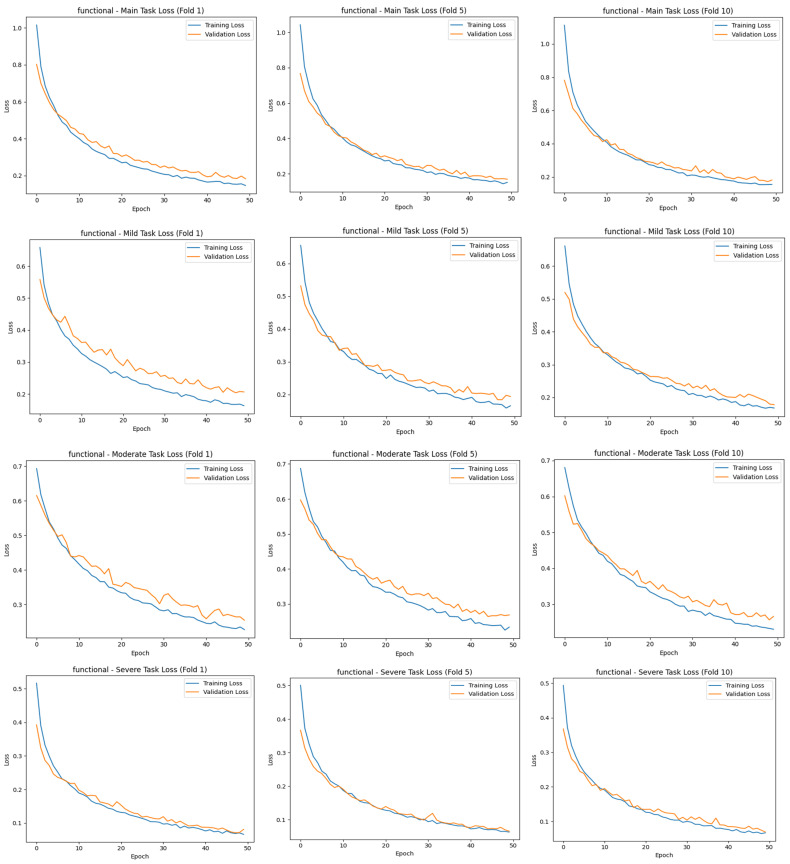
First-, middle-, and last-fold training and validation loss graphs for the MTL task.

A similar increase in accuracy is observed in the sub-tasks of mild, moderate, and severe. In the severe task, accuracy rates exceed 95%, while in the moderate task, accuracy is lower than in the others, and there are slight fluctuations in the validation curve. This suggests that the moderate class overlaps more with the other classes and is more difficult to distinguish. Overall, the graphs show that the model successfully manages the learning process across all tasks and exhibits a balanced performance between training and validation data, and the MTL structure enables efficient information sharing across tasks. Figure 11 shows the first-, middle-, and last-fold training and validation loss plots for the MTL task.

Figure 11 shows that for all tasks, training and validation losses decrease steadily as the epochs progress, reaching very low values around epoch 50. This trend shows that the model learns consistently on the tasks, and the optimization process is successful. Especially for the main task and the “severe” subtask, training and validation losses decreased almost in parallel and did not show excessive divergence along the graphs, indicating that the model avoided overfitting in these tasks. In the “moderate” and “mild” tasks, the verification loss was sometimes higher than the training loss in some folds, but this difference was generally controlled, and no serious overfitting problem was observed. Overall, the graphs show that the model performs well on all tasks, successfully maintaining the balance between training and validation processes. The confusion matrix scores of precision and recall for the MTL task were also obtained and are presented in Table 7. These scores, obtained by a 10-fold CV on the training and validation sets, show the extent to which the model can correctly identify each class and avoid false positive/negative classifications.

**Figure 12:**
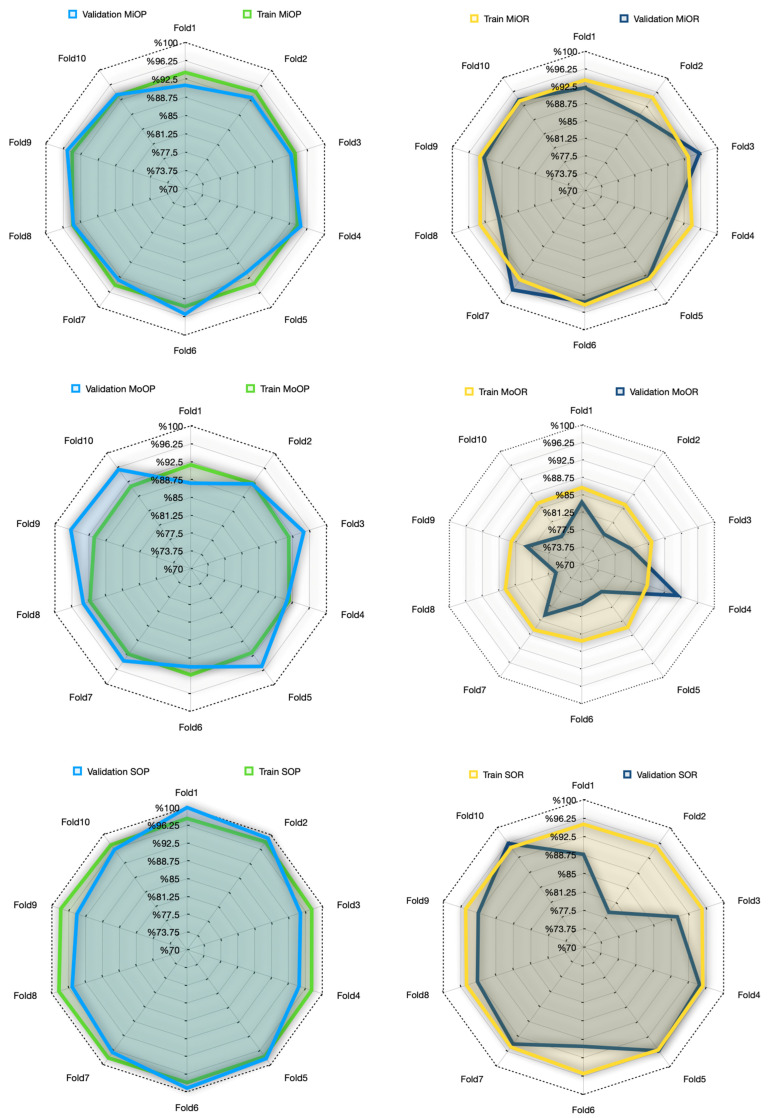
Comparative precision and recall rates for cirrhosis stages.

When Table 7 is analyzed, it is seen that the precision values in the validation set for the mild stage range between 91.2% and 95.7%, while the recall values range between 89.4% and 96.4%. This finding shows that the model is both accurate and balanced in recognizing this stage. Although the precision scores for the moderate stage were high (87.9–96.5%), the recall scores were more variable and relatively low (75.8–91.7%). This suggests that the model sometimes has difficulty in recognizing moderate cases and may tend to confuse this stage with other stages. Severe was the stage with the highest performance in both the training and validation sets. In the validation set, precision values ranged between 94.4% and 100%, and recall values ranged between 78.7% and 96.1%. These higher scores suggest that the severe stage may have more visually salient features and be more reliably identified by the model. Overall, these results show that the MTL architecture can learn discriminative representations for each stage and provide an explainable, balanced classification performance. Comparative precision and recall plots for each phase of the MTL task are presented in Figure 12.

As shown in Figure 12, the precision values for the mild and severe stages are quite consistent across the training and validation sets, showing high reliability with values above 90%. Especially in the severe stage, it is noteworthy that the model is consistent and highly successful in both sets, with no large deviations between folds. This can be attributed to the fact that the severe stage has more prominent visual features and is learned by the model with strong representations. A similar trend was observed for the mild stage; the variations in the validation data were limited and supported the overall model stability. On the other hand, significant fluctuations were observed in the moderate stage, especially in the recall values. In the validation MoOR (Moderate Output Recall) graph, some folds dropped as low as 70%, indicating that the model is unstable in its capacity to correctly recall this class. This fluctuation can be explained by the fact that the moderate population is more visually ambiguous, with high transitivity between classes. Although the MoOR values in the training data are relatively stable, this volatility in the validation data relatively weakens the generalization success of the moderate stage. This finding suggests that for the MTL model to better discriminate the moderate stage, the sample diversity should be increased or supported by XAI methods.

### 3.4. Explainable Artificial Intelligence with Grad-CAM

**Figure 13:**
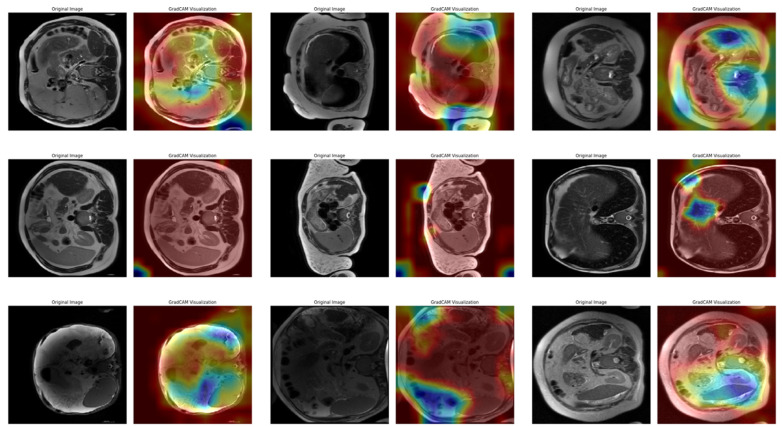
Visual comparison of heat maps obtained by Grad-CAM method. Each row contains three sample images for the mild, moderate, and severe classes, respectively. In the images, the left columns represent the original images, and the right columns represent the Grad-CAM outputs showing the regions that the model pays the most attention to when making decisions.

The Grad-CAM method has been applied to many different fields in recent years and is effective in improving model explainability, and medical image analysis is one of these fields. Therefore, in this study, the Grad-CAM method was used to explain the decision processes of the model and determine which features are effective in classification. Figure 13 shows three sample heatmaps from the first fold of the 10-fold CV (the fold with the lowest accuracy rate of 95.08%) obtained from the Grad-CAM application for each of the three stages.

**Figure 14:**
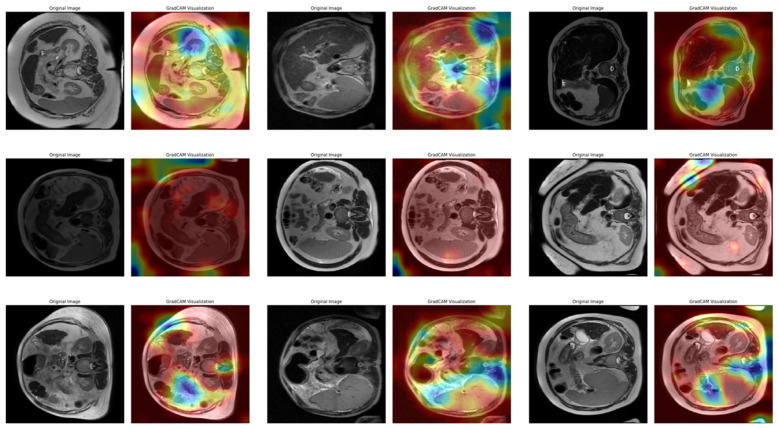
Examples of heatmaps obtained by Grad-CAM method for Fold 4. Each row contains three images for the mild, moderate, and severe classes, respectively. The left columns contain the original MR images, and the right columns contain the Grad-CAM visualizations of the regions that the model pays attention to during classification.

In Figure 13, a total of nine images, each consisting of three examples of three different stages (mild, moderate, or severe), are listed row by row. Each pair of images contains the original image and the Grad-CAM output, which shows the regions where the model concentrates when making decisions. In the first row of examples belonging to the mild class, the model’s attention is mostly distributed in the outer regions of the images. In the heatmaps, high intensity (warm colors) is located near the edges of the image. This suggests that the model considers more superficial structures when defining this class. In the moderate class in the second row, the distribution of attention appears to be more dispersed and spread over a larger area. In the images, it is clear that the model is unable to identify a clear focal point and receives information from multiple regions in the decision process. This suggests that the moderate class has structurally fewer clear boundaries and contains more uncertainty in terms of classification. In the examples of the severe class in the third row, the attention maps are oriented toward more centralized and distinct areas. It is seen that the model focuses more clearly on certain regions and makes decisions based on these regions. Since the attention intensity is more consistent for this class, it can be said that the model performs the classification more consistently. Figure 14 shows three sample heatmaps from Fold 4 of the 10-fold CV (the fold with an intermediate accuracy rate of 96.41%) obtained from the Grad-CAM application for each of the three stages.

**Figure 15:**
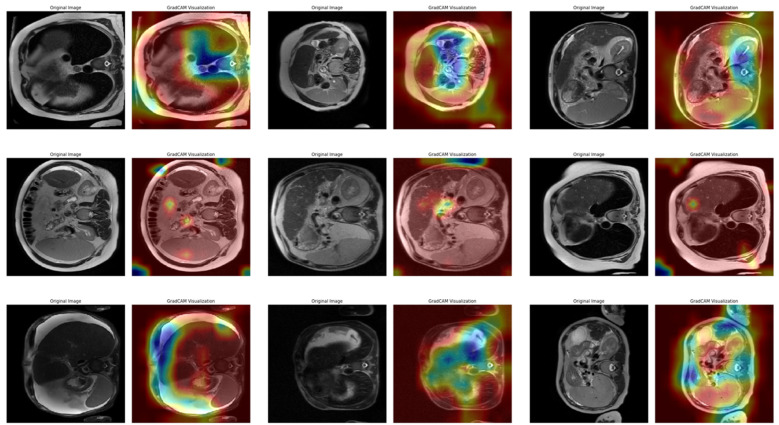
Examples of heat maps obtained by Grad-CAM method for Fold 10. Each row contains three images belonging to the mild, moderate, and severe classes, respectively. The left columns contain the original images, and the right columns contain the Grad-CAM visualizations showing the spatial areas that the model pays attention to during the decision process.

Figure 14 shows the Grad-CAM output of the fourth fold after classification with the VGG16 model. This fold is a fold with a 96.41% accuracy rate, which is an intermediate performance in the cross-validation set. The images are organized as three examples for each cirrhosis stage, with each row representing mild, moderate, and severe classes, respectively. In the mild class examples in the first row, the model’s attention distribution is more widely distributed across the image. In the heatmaps, intense activations appear to occur in multiple areas rather than focusing on a single region. This suggests that the model considers various structural cues in low-stage classifications and draws information from more regions in the decision process. In the second row of the moderate class, the attention maps contain more irregular and localized activations. In some cases, high activations can also be seen in the corner regions of the image or non-liver areas. This suggests that the model still experiences decision ambiguity in the moderate classification and that visual complexity may be effective at this stage. In the third row of the severe class, the attention maps appear to be more clearly focused on specific areas. Hot regions are usually concentrated in the center of the image or certain structural regions. This suggests that the model develops a more stable attention mechanism in the decision process for advanced classes and that class discrimination can be made more clearly at this stage. Figure 15 shows three sample heatmaps for each of the three stages from Fold 10 of the 10-fold CV (the fold with the highest accuracy rate of 97.91%) obtained from Grad-CAM.

In Figure 15, in the first row of examples belonging to the mild class, the model’s attentional distribution became more pronounced compared to the previous folds. The activations were focused on more limited regions. This suggests that the model developed a more stable and selective attentional mechanism when making decisions in low-stage samples. In the moderate class in the second row, more coherent and focused heat maps are observed compared to the scattered attention profile in the previous folds. The model draws information from fewer regions during decision-making, which contributes to classification accuracy. In the images, it is observed that the attention distribution is concentrated in certain regions and is far from random. In the examples in the third row of the severe class, the model’s attention is again centralized and focused on specific areas, consistent with high accuracy. In the heatmaps, it is noticeable that the model attributes high importance to only a few regions in its decision-making and that these regions are consistent across the examples. Overall, this fold shows that the model can make clearer distinctions between classes and that Grad-CAM outputs are the highest in terms of visual consistency. This suggests that the decision logic is less random and more systematic in scenarios where the model works with high accuracy. The focus of attention distribution for each class and the overall decision consistency of the model were technically evaluated and are presented in Table 8.

These findings show that the visualizations obtained with the Grad-CAM method are effective in explaining the decision logic of the model. Through the visuals, it was possible to analyze the regions from which the model extracted information and the differences in attention distribution between classes could be interpreted from a technical point of view. In this context, explainable artificial intelligence methods provide an important tool for increasing model transparency and evaluating reliability as well as accuracy.

## 4. Discussion

ML and DL studies on the detection of liver cirrhosis and related diseases in the literature were summarized based on the datasets, algorithms, accuracy rates, and advantages/disadvantages used and are presented in Table 9.

The hybrid CNN-, stacked ensemble-, and XAI-based approach presented in this study offers significant advantages in the diagnosis and staging of liver cirrhosis compared to existing methods in the literature. Most of the studies summarized in Table 9 reported accuracy rates in the range of 88–97% using laboratory parameters or limited image data. For example, ref. [16] achieved 97% accuracy with RF, while [20] reported a high performance of 99.3% with Capsule Net on MR images. However, most of these studies either focus on a single algorithm or have questionable generalization capabilities due to limited data diversity. In this study, the stacked ensemble method developed using a heterogeneous and comprehensive dataset, such as CirrMRI600+, achieved 96.92% accuracy in binary classification and 96.45% accuracy in stage detection with VGG16. These results significantly exceed the 75% accuracy rates reported in MRI-based studies such as [21], strengthening the potential of pre-trained DL models in clinical applications.

One of the most important contributions of this study is the integration of MTL and XAI to increase model transparency and clinical usability. Most studies in the literature (e.g., [18]) fail to explain the decision processes of their models despite their high accuracy. In this study approach, Grad-CAM-generated heat maps show that the model focused on anatomically significant regions of the liver, especially in the severe stage. This finding is a critical advantage that may reduce clinicians’ doubts regarding model reliability. Moreover, this study model, which learns from inter-stage associations with MTL, exhibited an outstanding discrimination performance of 99.7% AUC. This result is superior to the 97.6% accuracy reported by [23] and overcomes the generalization problems encountered in models working with limited data (e.g., [22]).

With these results, answers to the RQs mentioned in the Introduction section were obtained. Successful detection of liver cirrhosis is possible with DNNs (RQ1). The stacked ensemble approach in this study met this target with 96.92% accuracy, outperforming traditional ML methods in the literature (e.g., [17] 94%). In disease staging, the VGG16-based model achieved 96.45% accuracy, proving the superiority of DL in this task (RQ2), especially compared to the 75% result [21]. Grad-CAM applications improved clinical reliability by visualizing the consistency of model decisions with anatomical regions in MR images (RQ3). This offers an important alternative to studies with high accuracy but lacking transparency, such as [20]. A recent study by the authors of [37] evaluated various methods of liver measurement, highlighting the accuracy and reliability of volumetric segmentation based on computed tomography. While their approach focuses on CT imaging, this study leverages MRI data and DL techniques to estimate liver cirrhosis stages. This contrast underscores the potential of MRI-based DL models in providing non-invasive and accurate staging of liver diseases.

This study also has some limitations. In particular, the relatively small number of patients in the severe class (73 patients) caused the recall values of the model to decrease in this group. This is a problem also encountered in similar studies in the literature (e.g., [19]). The staging dataset exhibited class imbalance; however, data augmentation or re-weighting strategies were not applied, as the aim was to build a model trained exclusively on real, non-synthetic MRI data. This choice was made to ensure maximum clinical fidelity, though future work may explore synthetic augmentation to improve model performance for under-represented stages. Additionally, although images were acquired from three different MRI scanners, explicit domain adaptation or harmonization techniques (e.g., ComBat or CycleGAN) were not applied to mitigate device-specific signal variability. Instead, general preprocessing procedures, such as intensity normalization and resizing, were used. Future studies could benefit from scanner-specific harmonization to further improve model generalizability across multi-center datasets. Furthermore, in the current implementation, image slices were randomly divided into training and validation folds without explicit patient-wise separation. Since the dataset was organized at the slice level and lacked consistent patient identifiers across files, strict patient-level partitioning could not be ensured. It is acknowledged that this limitation may result in partial patient overlap across folds, potentially leading to an optimistic estimation of validation performance. This issue has been noted and is planned to be addressed in future work by incorporating patient-level data structures and identifiers. One another limitation of this study is the absence of expert validation for both the model’s predictions and its visual explanations. Although Grad-CAM heatmaps were employed to highlight class-discriminative regions in the MRI images, these visualizations were not independently reviewed by a radiologist, and thus their clinical interpretability remains unverified. Similarly, the model’s classification and staging outputs were not directly compared with expert radiologist assessments, which limits the ability to gauge clinical alignment. Future work will address these issues by incorporating radiological evaluation to benchmark both the model’s predictions and the interpretability of its attention maps, thereby enhancing the clinical relevance of the approach.

Nevertheless, the stacked ensemble- and MTL-based framework presented in this study fills the gap in the literature not only with high accuracy rates but also with explainability and clinical relevance. This approach can be used as a practical tool to support radiologists, especially in the diagnosis of early-stage cirrhosis.

## 5. Conclusions

This study presents a comprehensive framework for the automatic diagnosis and staging of liver cirrhosis using MR images. Integrating hybrid CNN architectures, stacked ensemble learning, MTL, and XAI techniques, this approach achieves robust performance, outperforming many existing methods in the literature, with 96.92% accuracy in binary classification and 96.45% accuracy in stage prediction. The use of Grad-CAM for interpretability provides clinical confidence by visually validating model decisions against radiological features, especially in severe cirrhosis cases where anatomical changes are evident. Furthermore, MTL demonstrates exceptional generalization (AUC: 99.7%) by leveraging shared representations across cirrhosis stages, addressing class imbalance issues common to medical imaging datasets.

While the results are promising, this study has several limitations. No expert radiologist review was conducted for either the model predictions or the Grad-CAM visualizations, limiting clinical interpretability. Additionally, patient-wise separation was not implemented during cross-validation, which may have introduced optimistic bias in performance estimates.

This work establishes a new benchmark for explainable, end-to-end cirrhosis diagnosis and bridges the gap between AI performance and clinical usability. The adaptability of the framework suggests potential applications in other fibrotic liver diseases and paves the way for AI-enabled radiology workflows.

The main contributions of the study to the field (employing classification and staging liver cirrhosis) can be listed as follows:

- A novel stacked ensemble combining CNN feature extractors with XGBoost meta-learning;
- Clinically validated interpretability via Grad-CAM;
- First-of-its-kind MTL for cirrhosis staging.

Suggestions for future research can be stated as follows: Using the gender information available in the current dataset, studies can be conducted to examine the variation of cirrhosis markers and model performance by gender. This will contribute to the development of personalized diagnostic models. The MTL performance of different pre-trained models (such as ResNet152 and EfficientNetV2-L) can be compared. For failed modules (EfficientNet series), alternative architectures or hyperparameter optimizations can be tried. Deeper variants of successful models (such as VGG19 and DenseNet169) can be tested. Transfer learning strategies can be reviewed, especially for underperforming modules. Data augmentation techniques and class balancing methods can be used to improve the performance of minority classes. Expanded studies, in line with these recommendations, will accelerate the integration of AI-assisted cirrhosis diagnosis systems into clinical applications and contribute to personalized medicine approaches. The XAI framework presented in this study provides a model that can be applied in the diagnosis of similar fibrotic liver diseases. This study highlights how hybrid AI systems can improve diagnostic accuracy while maintaining transparency, which is a critical step toward the adoption of responsible AI in hepatology.

## Figures and Tables

**Table 1:** Dataset gender and age distribution.

Category	Healthy Group	Cirrhosis Group		
		Mild	Moderate	Severe
Total Subjects	55	131	114	73
Female	34	54	47	27
Male	21	77	67	46
Mean Age	62.78 ± 14.93	59.56 ± 13.92	60.22 ± 14.93	60.88 ± 11.51

**Table 2:** Data information after preprocessing.

Class	Stage	Patient Number	Total Images
Healthy	-	55	2143
Cirrhosis	Mild	131	2838
	Moderate	114	2391
	Severe	73	1473

**Table 3:** Modules and models used in the study.

No.	Module	Model	N	Module	Model
1	xception	Xception	8	inception_resnet_v2	InceptionResNetV2
2	vgg16	VGG16	9	densenet	DenseNet121
3	resnet	ResNet50	10	efficientnet	EfficientNetB0
4	resnet_v2	ResNet50V2	11	efficientnet_v2	EfficientNetV2B0
5	nasnet	NASNetMobile	12	mobilenet	MobileNet
6	convnext	ConvNeXtTiny	13	mobilenet_v2	MobileNetV2
7	inception_v3	InceptionV3	14	mobilenet_v3	MobileNetV3Small

**Table 4:** The 10-fold CV results for the stacked ensemble method.

Class	Sup.	Metric	Fold 1	Fold 2	Fold 3	Fold 4	Fold 5	Fold 6	Fold 7	Fold 8	Fold 9	Fold 10	Mean	Std. Dev.
0_health	215	precision	0.96	0.94	0.94	0.98	0.95	0.95	0.96	0.95	0.93	0.95	0.9512	0.0119
		recall	1.00	1.00	0.98	0.99	0.99	0.98	1.00	0.98	1.00	0.99	0.9893	0.0072
		f1-score	0.98	0.97	0.96	0.98	0.97	0.96	0.98	0.96	0.97	0.97	0.9698	0.0075
1_cirrhosis	214	precision	1.00	1.00	0.98	0.99	0.99	0.98	1.00	0.98	1.00	0.99	0.9889	0.0075
		recall	0.96	0.94	0.93	0.98	0.95	0.94	0.96	0.94	0.93	0.95	0.9491	0.0130
		f1-score	0.98	0.97	0.96	0.98	0.97	0.96	0.98	0.96	0.96	0.97	0.9685	0.0080
General	429	accuracy	0.98	0.97	0.96	0.98	0.97	0.96	0.98	0.96	0.96	0.97	0.9692	0.0077
		macro avg	0.98	0.97	0.96	0.98	0.97	0.96	0.98	0.96	0.96	0.97	0.9692	0.0077
		weighted avg	0.98	0.97	0.96	0.98	0.97	0.96	0.98	0.96	0.96	0.97	0.9692	0.0077

**Table 5:** Performance results for cirrhosis stages.

No.	Module	Model	Accuracy				Loss			
			First	Mid	Last	Mean	First	Mid	Last	Mean
1	xception	Xception	90.61%	91.80%	92.24%	91.39%	0.2969	0.2634	0.3018	0.2877
2	vgg16	VGG16	95.08%	96.87%	97.91%	96.45%	0.1346	0.1284	0.1064	0.1199
3	resnet	ResNet50	79.88%	75.82%	83.58%	80.47%	0.4787	0.4779	0.4256	0.4679
4	resnet_v2	ResNet50V2	94.04%	93.28%	94.48%	93.56%	0.3228	0.3188	0.2915	0.3766
5	nasnet	NASNetMobile	91.80%	91.79%	92.83%	91.84%	0.2762	0.2643	0.2042	0.2397
6	convnext	ConvNeXtTiny	90.01%	90.59%	91.04%	91.21%	0.2779	0.2358	0.2271	0.2294
7	inception_v3	InceptionV3	86.44%	82.84%	88.96%	86.17%	0.5372	0.6436	0.3622	0.5182
8	inception_resnet_v2	InceptionResNetV2	85.84%	88.06%	89.06%	88.31%	0.5143	0.3911	0.4401	0.4057
9	densenet	DenseNet121	94.19%	94.78%	93.44%	94.17%	0.2389	0.2236	0.1829	0.1817
10	efficientnet	EfficientNetB0	37.10%	42.69%	44.18%	41.84%	1.0571	1.0651	1.0652	1.0670
11	efficientnet_v2	EfficientNetV2B0	46.65%	49.78%	42.69%	45.80%	1.0148	1.0053	1.0372	1.0207
12	mobilenet	MobileNet	95.53%	94.63%	97.16%	95.91%	0.3331	0.3506	0.2041	0.2488
13	mobilenet_v2	MobileNetV2	94.33%	95.82%	95.22%	95.69%	0.3151	0.1747	0.2203	0.1962
14	mobilenet_v3	MobileNetV3Small	66.76%	67.01%	73.13%	67.99%	0.7463	0.7209	0.6885	0.7141

**Table 6:** MTL 10-fold CV results.

Dataset	Metric	Fold1	Fold2	Fold3	Fold4	Fold5	Fold6	Fold7	Fold8	Fold9	Fold10
Train	MaOAcc%	96.17	96.58	95.65	95.98	95.99	96.37	96.55	96.15	96.27	95.50
	MaOLoss	0.1460	0.1386	0.1557	0.1483	0.1508	0.1425	0.1409	0.1461	0.1498	0.1548
	MaOAUC%	99.69	99.75	99.59	99.69	99.65	99.74	99.74	99.70	99.68	99.59
	MiOAcc%	94.78	95.47	94.57	95.07	94.82	95.27	95.03	94.87	95.03	94.75
	MiOLoss	0.1633	0.1524	0.1693	0.1645	0.1657	0.1596	0.1631	0.1619	0.1652	0.1679
	MoOAcc%	92.57	92.62	92.15	92.05	92.43	92.75	92.82	92.95	91.89	92.18
	MoOLoss	0.2281	0.2238	0.2319	0.2297	0.2336	0.2248	0.2239	0.2263	0.2363	0.2304
	SOAcc%	98.38	98.52	98.48	98.48	98.63	98.59	98.58	98.57	98.55	98.32
	SOLoss	0.0659	0.0627	0.0654	0.0654	0.0633	0.0648	0.0617	0.0648	0.0634	0.0669
	Loss	0.3745	0.3581	0.3894	0.3786	0.3809	0.3668	0.3654	0.3729	0.3819	0.3877
Validation	MaOAcc%	93.74	94.05	96.71	95.46	94.68	95.31	96.55	94.68	94.99	94.51
	MaOLoss	0.1829	0.1983	0.1562	0.1757	0.1687	0.1740	0.1466	0.1853	0.1759	0.1811
	MaOAUC%	99.45	99.20	99.75	99.43	99.58	99.55	99.81	99.61	99.49	99.41
	MiOAcc%	92.33	92.96	94.67	93.74	93.43	95.46	95.77	93.27	94.68	94.83
	MiOLoss	0.2064	0.1969	0.1710	0.1871	0.1941	0.1779	0.1576	0.1975	0.1796	0.1779
	MoOAcc%	90.77	89.36	92.32	93.27	89.98	90.29	92.32	89.20	93.58	90.44
	MoOLoss	0.2549	0.2776	0.2363	0.2461	0.2683	0.2557	0.2403	0.2724	0.2387	0.2661
	SOAcc%	97.65	95.46	97.02	98.12	98.90	97.65	97.81	97.49	97.03	98.43
	SOLoss	0.0807	0.1037	0.0736	0.0758	0.0660	0.0790	0.0730	0.0800	0.0865	0.0695
	Loss	0.4541	0.4874	0.3970	0.4302	0.4331	0.4304	0.3819	0.4604	0.4285	0.4375

MaOAcc = Main Output Accuracy, MaOLoss = Main Output Loss, MaOAUC = Main Output AUC, MiOAcc = Mild Output Accuracy, MiOLoss = Mild Output Loss, MoOAcc = Moderate Output Accuracy, MoOLoss = Moderate Output Loss, SOAcc = Severe Output Accuracy, SOLoss = Severe Output Loss.

**Table 7:** Precision and recall rates for 10-fold CV on training and validation sets.

Dataset	Metric	Fold1	Fold2	Fold3	Fold4	Fold5	Fold6	Fold7	Fold8	Fold9	Fold10
Train	MiOP	0.9386	0.9459	0.9368	0.9419	0.9404	0.9414	0.9444	0.9422	0.9434	0.9361
	MiOR	0.9382	0.9481	0.9345	0.9426	0.9355	0.9466	0.9377	0.9370	0.9374	0.9387
	MoOP	0.9186	0.9223	0.9167	0.9177	0.9187	0.9234	0.9220	0.9225	0.9131	0.9141
	MoOR	0.8655	0.8587	0.8581	0.8479	0.8679	0.8651	0.8747	0.8732	0.8598	0.8626
	SOP	0.9770	0.9811	0.9767	0.9764	0.9787	0.9805	0.9825	0.9844	0.9802	0.9733
	SOR	0.9501	0.9530	0.9538	0.9554	0.9589	0.9575	0.9515	0.9503	0.9523	0.9508
Validation	MiOP	0.9122	0.9310	0.9281	0.9490	0.9127	0.9573	0.9316	0.9407	0.9542	0.9384
	MiOR	0.9215	0.8999	0.9595	0.8996	0.9331	0.9406	0.9646	0.8947	0.9281	0.9418
	MoOP	0.8799	0.9204	0.9499	0.9134	0.9541	0.9066	0.9397	0.9372	0.9649	0.9579
	MoOR	0.8341	0.7806	0.8104	0.9169	0.7727	0.7857	0.8348	0.7585	0.8249	0.7745
	SOP	1.0	0.9905	0.9516	0.9487	0.9840	0.9923	0.9679	0.9549	0.9444	0.9609
	SOR	0.8889	0.7879	0.9008	0.9487	0.9609	0.9021	0.9438	0.9270	0.9252	0.9609

MiOP = Mild Output Precision, MiOR = Mild Output Recall, MoOP = Moderate Output Precision, MoOR = Moderate Output Recall, SOP = Severe Output Precision, and SOR = Severe Output Recall.

**Table 8:** Comparison of attention distribution of Grad-CAM heatmaps obtained at different 10-fold validation layers by class.

Fold No.	Acc. (%)	Mild-Stage Attention Distribution	Moderate-Stage Attention Distribution	Severe-Stage Attention Distribution	General Observation
1	95.08	Widely dispersed, more concentrated at the edges	Disorganized, focusing on too many different areas	Focus on clear, centralized regions	The model’s effort to identify the difference between classes is observed
4	96.41	There are widespread but clearer foci	Partially irregular, activation in corner regions	Concentrated, centralized attention in specific areas	The model’s decisions are becoming more consistent
10	97.91	More stable, focused on specific areas	Consistent focus on fewer regions	Similar attention patterns in consistent, in-class samples	The model makes more systematic decisions with the highest accuracy

**Table 9:** Literature studies on liver cirrhosis.

Author (Year)	Dataset	Model(s)/Algorithm	Findings	Advantages/Disadvantages
[11]	Patients’ laboratory parameters	DT, LD, SVM Fine Gaussian, LR	LD: 95.8% Acc.	A: High accuracy. D: Using only specific algorithms, generalizability may be limited.
[12]	UCI ML Repo	ANN	88.4% Acc.	A: Use of ANN. D: Relatively low Acc.
[13]	UCI data (from Kaggle)	LR, k-NN, XGBoost, SVM, Gaussian NB, RF, DT, GB, CatBoost, AdaBoost, LightGBM	RF: 88.55% Acc.	A: Comparison of many algorithms. D: Differences between accuracy rates are limited.
[14]	Hepatitis C-based data	NB, RF, k-NN, DT, DL	DL: 95.5%	A: High accuracy. D: The dataset is focused on a specific group of patients.
[15]	Hepatitis C-based data	RF	High prediction performance	A: Use of the ensemble learning technique. D: Focused on a specific group of patients.
[16]	Liver cirrhosis dataset	SVM, DT, RF	RF: 97% Acc.	A: High accuracy. D: Only certain algorithms are used.
[17]	Clinic + Lab. data	k-NN, SVM, NN	k-NN: 94% Acc.	A: Comparison of different algorithms. D: Differences between accuracy rates are limited.
[18]	Hepatitis C-based data	RNN vs. LR	Better performance with RNN	A: Superior performance of DL models. D: Model complexity and interpretability.
[19]	US dataset	CNN + SVM	90% Acc.	A: Image-based diagnostic system. D: Accuracy can be higher.
[20]	MRI	CNN + Capsule Net	99.3% Acc.	A: Very high accuracy. D: Model complexity and computational cost.
[21]	T2-weighted MRI	ResNet50, DenseNet121	ResNet50: 75% Acc.	A: Using pre-trained DL models. D: Relatively low accuracy.
[22]	ML dataset	DL (MLP), DT, KNN, LR, NB, RF, SVM	MLP: 80.48% Acc.	A: Comparison of different algorithms. D: Accuracy may be higher.
[23]	MRI (26 vs. 56 features)	DL	97.6% Acc. (56 features)	A: High accuracy rates. D: Increasing the number of features can increase the complexity of the model.

## Data Availability

The data used in this study are openly available at https://doi.org/10.17605/OSF.IO/CUK24.
